# Evaluation of the International Child Development Programme (ICDP) as a community-wide parenting programme

**DOI:** 10.1080/17405629.2013.793597

**Published:** 2013-05-10

**Authors:** Lorraine Sherr, Ane-Marthe Solheim Skar, Claudine Clucas, Stephen von Tetzchner, Karsten Hundeide

**Affiliations:** 1 Department of Infection & Population Health, University College London, London, UK; 2 Department of Psychology, University of Oslo, Oslo, Norway

**Keywords:** Early child development, Caregiver guidance, Community intervention, Evaluation, ICDP

## Abstract

**Background:**

Many parenting programmes lack proper evaluation, especially under community-wide implementation.

**Objective:**

Examining the effectiveness of the eight-week International Child Development Programme (ICDP), implemented as a general programme.

**Methodology:**

Non-clinical caregivers attending ICDP (*N* = 141) and a non-attending community comparison group (*N* = 79) completed questionnaires on parenting, psychosocial functioning, and child difficulties before and after ICDP course. Analyses compare changes in scores for both groups over time.

**Results:**

The ICDP group showed more positive attitudes towards child management and reported better child management, improved parental strategies and less impact of child difficulties. Caregivers with low initial scores benefited most. The comparison group showed little change with a significant decrease in scores on the caregiver–child activity scale.

**Discussion:**

The results suggest that caregivers in the community who do not show clinical signs or have children with behaviour or other disorders, may benefit from participating in parent training based on ICDP.

## INTRODUCTION

Throughout the history of child-care, there has been a focus on the importance of parent–child relations and early child development (von Tetzchner, 2012). In most societies, parents are the core of children's early social environment, and parenting strategies and parent–child relations are assumed to have an impact on all aspects of child development (Carlo, Mestre, Samper, Tur, & Armenta, 2011; Landry, Smith, & Swank, 2003; Sandler, Schoenfelder, Wolchik, & Mackinnon, 2011). Interventions aimed at supporting parenting have mainly been directed at families at risk, such as socially disadvantaged families (e.g., Hutchings et al., 2007; McDonald, FitzRoy, Fuchs, Fooken, & Klasen, 2012; Scott, O'Connor, et al., 2010) and those with clinical diagnoses (e.g., Law, Plunkett, Taylor, & Gunning, 2009; Webster-Stratton, Kolpacoff, & Hollingsworth, 1988). Boyd & Gillham (2009) found more positive parent–child interactions and better coping skills in the children of depressed parents following intervention. Parents of children with attention-deficit hyperactivity disorder (ADHD) reported increased confidence and less stress after training (Anastopoulos, Shelton, DuPaul, & Guevremont, 1993). Parental training is also found effective in reducing children's conduct problems (e.g., Ogden & Hagen, 2008; Scott, Sylva, et al., 2010).

Challenges experienced in raising children are common and the positive results of parenting programmes with clinical groups may benefit a broader group of caregivers (Rodrigo, Almeida, Spiel, & Koops, 2012) to improve all aspects of caregiving with possible onward benefits on child behaviour and caregiver mental health (Long, 2007; Sanders & Morawska, 2010). One of the few efficacy studies of a generally implemented programme found increased positive and reduced negative parenting behaviour (Hahlweg, Heinrichs, Kuschel, Bertram, & Naumann, 2010).

Yet there is a lack of efficacy research evidence (Rodrigo et al., 2012 Scott, 2010). In a review of 46 general and targeted interventions, Sandler et al. (2011) found long-term effects of parenting interventions on child development and behaviour, but a lack of explanation of the processes mediating these effects. The effects of programmes designed for specific clinical groups may be easier to explain but may not apply to caregivers with more ordinary challenges.

In order to develop optimal programmes for community-wide implementation, there is a need to investigate programmes based on different theoretical foundations and which comprise a variety of elements. Theoretically, most current parenting programmes are based on social learning theory and behaviour change, e.g., *The Incredible Years* (Webster-Stratton et al., 1988), *Parental Management Training* (Patterson, 2002) and the Triple P-programme (Sanders, 2008). Few evaluations are conducted in community settings (Hutchings et al., 2011). ICDP is a non-instructive psychosocial intervention programme directed towards parents and other caregivers. The programme is well recognized and is used in about 35 countries, including both socially well-functioning societies and societies where political unrest and war may make parenting especially challenging, and in collaboration with organizations such as *Save the Children, Unicef, Care*, and WHO. However, there is no evaluation of ICDP implemented as a community-wide programme in a socially well-functioning society. The present study examined the impact of ICDP courses on a general community sample of caregivers in Norway. The main research question was whether participation in an ICDP course would have a positive impact on parenting strategies and on how caregivers perceived their children and themselves. The moderating effects of self-efficacy, depression and social support were also investigated.

## METHOD

The study used a two-group design with a natural intervention group (*N* = 141) and a comparison group (*N* = 79) who both completed questionnaires before and after the intervention group's ICDP course.

### The ICDP programme: Content and implementation

The theoretical foundation of ICDP is derived from developmental and humanistic psychology with focus on sensitive adult adjustment and empathy (Hundeide & Rye, 2010). It is non-instructive and aims to guide carers’ understanding of their children and interaction with them. The philosophy is formulated in three dialogues containing eight guidelines: the emotional dialogue (e.g., showing loving feelings, praising and acknowledging the child), the comprehension dialogue (e.g., supporting the child's meaning-making and showing enthusiasm for the child's experiences), and the regulative dialogue (e.g., regulating the child's actions step-by-step; Hundeide, 2001; see http://icdp.info for details on ICDP).

ICDP courses are offered nationally in Norway by the Ministry of Children, Equality and Social Inclusion through “The Parental Guidance Programme”. A filter-down approach is applied where the facilitators become qualified to run caregiver groups, and some facilitators proceed to become qualified to train new facilitators. Mothers and fathers of children at all ages may participate, but ICDP groups tend to contain parents with children in the pre- school age, and are usually delivered through ICDP educated staff at kindergartens and child health centres. The groups usually consist of 5–10 caregivers attending eight weekly two-hour sessions, one meeting for each guideline. Caregivers take an active role, participate in group discussions, role play the guidelines, and do home assignments, like “Try to follow your child's lead. What happens?” The facilitators give positive comments, encourage active involvement and facilitate discussions. Further details at http://www.bufetat.no/foreldrerettleiing/.

### Participants

All ICDP facilitators were contacted and logged forthcoming groups for potential inclusion (Sherr, Skar, Clucas, von Tetzchner, & Hundeide, 2009). A total of 75 ICDP groups were approached during the data collection period. The groups were run at kindergartens and health centres with carers recruited through open billboard information, staff advertisement or invitation. At the first meeting, caregivers were informed about the project verbally and in writing.

A group of 269 caregivers completed the first set of questionnaires and 141 (52.4%) completed follow-up questionnaires. A comparison group (*N* = 157) to control for passage of time was recruited from child health centres and kindergartens in areas where the ICDP programme was not implemented, of whom 79 (50.3%) returned the second questionnaire. The data were collected from October 2008 to March 2010. The ICDP caregivers had an average age of 36.6 (range 23–60), 2.0 children (range 1–6) and 3.6 people in the home (range 1–6). The focus child was 4.0 years on average (*SD* = 2.64, range 0.5–16), 66 girls and 59 boys (16 caregivers did not provide information about gender). Caregivers in the comparison group had an average age of 34.2 (*SD* = 1.83, range 24–47). They had an average of 1.8 children (range 1–4) and 3.5 people in the home (range 1–6). The focus child was 3.3 years (*SD* = 1.83, range 0.25–11), 35 were girls and 26 boys (18 caregivers did not provide information about gender). Caregivers in the two groups did not differ significantly on these variables. Caregivers in the comparison group were significantly more likely to be married or live with a partner and to have higher education than the ICDP group but the groups did not differ on gender, being born in Norway or employment (see Table 1).

**TABLE 1:** Characteristics of caregivers in the ICDP group and the comparison group

*Categorical variables*		*ICDP (%)*	*Comparison (%)*	*χ*^2^	*p*
*Gender*	Males	25.5	21.5	0.38	.536
	Females	74.5	77.2		
*Education*	Higher education	58.9	74.7	5.34	.019*
	No higher education (high school or less or other after high school)	41.1	25.3		
*Born in Norway*	Yes	90.8	89.9	0.004	.952
	No	9.2	8.9		
*Civil status*	Married/partner	89.4	94.9	3.99	.042*
	Separated/divorced/widow/single	9.9	2.5		
*Employment*	Full time	55.3	70.9	5.59	.061
	Part time	15.6	8.9		
	Other (e.g., at home or on leave)	26.9	17.7		

*Notes*: ICDP *N* = 141; Comparison *N* = 79. *p < .05.

### Materials

All participants completed a questionnaire designed to gather information about demographics, social relationships, and emotional and parenting issues. Measures include self-efficacy, depression, social support, parenting (activities with the child, discipline, household commotion, happiness with partner, parenting strategy, engagement with the child and child management). Child measures include the Strengths and Difficulties questionnaire (see Table 2).

**TABLE 2:** Study measurements

*Measure*	*Detail*
*Activities with the child*:	
The Parent–Child Activity Scale (Bigner, 1977)	Twenty-five items scored on a Likert scale 1 (*Never*) to 5 (*Always*), total scores ranging from 25 to 125. Cronbach's (α = .88 at first and .92 at second completion
*Positive discipline*:	
Conflict Tactics Scale (Straus, 1979)	Seven items on positive discipline created, e.g., “Praised them for achieving something on their own” and “Told them that you were proud of them”. Caregivers indicated how frequently they engaged in the behaviours (0, 1–2, 3–10 or more than 10 times). A summed score was created by adding midpoints for the response categories, ranging from 0 to 105. Cronbach's (α = .68 at first and .37 at second completion. (The full Conflict Tactics Scale was used, but the results for the other subscales are not reported due to poor inter-item reliability; Cronbach's (< .5)
*Household commotion*:	
The Confusion, Hubbub, and Order Scale (Matheny et al., 1995)	Fifteen items, which are scored as true or false, with summed scores ranging from 0 to 15. A higher score represents a more chaotic, disorganized and hurried household. Cronbach's (α = .73 at first and .73 at second completion
*Parenting strategy*:	
	“Parenting strategies” measured the parental strategies with a focus on the comprehensive dialogue in the ICDP components. The five items loaded on one factor at first completion. The summed score for “Parenting strategies” ranged from 5 to 30 (α = .72 at first and .76 at second completion). Negatively phrased items were reverse coded such that a higher score was always better
*Child management*:	
	“Child Management” measured child management strategies with a focus on the emotional and regulative dialogue in the ICDP. The scale consists of 22 items scored on a Likert scale from 1 *(Agree completely*) to 5 (*Completely disagree*). Average scores range from 1 to 5 (α = .77 at first and .69 at second completion). Negatively phrased items reverse coded, so a lower score was always better
*Engagement with the child*:	
	Ten bipolar items to measure key ICDP components, scored in counterbalanced order from 1 to 7. Three scales were created: “engagement scale” eight items (e.g., sensitive–insensitive), loading on one factor at first completion in a principal components analysis (PCA); “emotional engagement scale” six items (e.g., loving– unloving), loading on one factor at second completion, and a “strategic engagement scale” three items (e.g., rewarding–punitive), loading on one factor at second completion. One item (strict–lenient) did not load on any of the two factors at second completion. Three mean scores generate dranging from 1 to 7. For “engagement scale” Cronbach's (α = .86 at first and .84 at second completion; for “strategic engagement scale” (α = .72 at first and .76 at second completion; and for “emotional engagement scale” (α = .84 at first and .81 at second completion. A lower score indicates greater engagement
*Happiness with partner*:	
Drawn from the Dyadic Adjustment Scale (Spanier, 1976) *Strength and Difficulties: Questionnaire*	A visual analogue scale scored from 0 (*Extremely unhappy*) to 6 (*Perfectly happy*) was utilized
(SDQ; Goodman, 1999)	Five subscales (emotional symptoms, conduct problems, hyperactivity, peer problems, prosocial) generating three scores (1) total difficulties (0 to 40; (α = .73 at first and .74 at second completion), (2) prosocial scale score (0 to 10; (α = .75 at first and .80 at second completion), and (3) an impact score derived from questions on overall distress and social impairment ranging from 0 to 10
*Depression*:	
The Hospital Anxiety and Depression Scale (HADS; Zigmond & Snaith, 1983)	Consists of seven anxiety and seven depression items, which are scored from 0 (*Not at all*) to 3 (*Very often, most of the time, definitely, very much*), giving a summed score for depression ranging from 0 to 21 (α = .69 at first completion)
*Social support*:	
The Social Support Questionnaire – Short Form (SSQ6; Sarason et al., 1987)	Two scores were generated from this scale: a total number of social supports score ranging from 0 to 9 (α = .93 at first completion) and a satisfaction with social supports score ranging from 1 to 6 (α = .91 at first completion)

### Procedure

Caregivers completed the questionnaires at the first meeting. They were asked to complete a second questionnaire after the last group meeting or returned it by mail.

Caregivers in the comparison group received the first questionnaires at time of consent and the follow-up questionnaire by mail after the same number of weeks as the ICDP group. If the questionnaire was not returned, one reminder was sent.

### Plan of analyses

Chi-squared tests and *t*-tests were used to compare the groups on demographic variables, and to compare those lost to follow-up on demographic variables and scale scores to examine factors associated with no follow-up. As a result of differences between the ICDP group and the comparison in education, the study used 2 (Group: ICDP/comparison) × 2 (Education: higher education/not higher education) × 2 (Time of Measurement: before/after) mixed analysis of variance (ANOVA) with repeated measures on time of measurement. Interactions between Group and Time of Measurement are reported as these indicate differential changes for the intervention group and the comparison group and suggest an effect of the intervention on the outcome. Civil status differed between the groups but it was not possible to enter this factor in the main analysis because of the small number of caregivers who were not married or with a partner.

Two 2 (Group: ICDP/comparison) × 2 (Education: higher education/not higher education) × 2 (Time of Measurement: before/after) multivariate analyses of variance (MANOVAs) were used to study the effect of the intervention on the SDQ subscales (SDQ total difficulties and SDQ prosocial) and the subscales of the engagement scale (emotional engagement and strategic engagement).

In order to study whether the intervention had a differential effect according to carer self-efficacy, depression or social support, moderation analyses were conducted. A median split was used to categorize carers into low and high selfefficacy, low and high depression and low and high satisfaction with social support. This factor was added to the 2 (Group: ICDP/comparison) × 2 (Education: higher education/not higher education) × 2 (Time of Measurement: before/after) mixed ANOVA (or MANOVA).

### Ethical considerations

The study was approved by the Regional Committee for Medical and Health Research Ethics and the Norwegian Social Science Data Services. Therapist referral was available but no participants expressed a need for such a contact.

## RESULTS

### Attendance

In the ICDP group, 96 caregivers (68.1%) reported being offered eight meetings and 31 (21.9) were offered another number of meetings (14 missing). Forty-eight (34.0%) attended all meetings held, 37 (26.2%) missed one meeting, 22 (15.6%) missed two meetings and 13 (9.2%) missed more than two meetings (21 missing). Linear regression analyses showed no significant relationship between the number of sessions attended and change scores between first and second completion of questionnaires, when adjusting and when not adjusting for the number of meetings held.

### Follow-up

Compared to the initial ICDP group, participants who completed the post questionnaires were more likely to be married/cohabiting (90% vs. 79.8%), χ^2^ (1, 26) = 5.40, *p* = .02. They also reported significantly less depression (*M* = 3.18, *SD* = 2.33) than participants who did not complete the questionnaire after the course (*M* = 3.86, *SD* = 2.84), *t*(1, 25) = −2.09, *p* = .04). Compared to the initial group, participants in the comparison group who completed the second set of questionnaires were less likely to have a computer in the child's room (0% vs. 6.6%) *χ*^2^ (1, 155) = 5.37, *p* = .02.

### Parental behaviours and impact of child difficulties

Table 3 shows key variables for ICDP and comparison groups before and after the ICDP course. There was a significant interaction between Group and Time for parenting strategies, indicating that ICDP attendees improved their parenting strategies (*M* = 22.67 and 23.52), while the comparison group did not change or had slightly lower scores (*M* = 23.54 and 23.30). Follow-up analyses confirmed a significant improvement in the ICDP group on parenting strategies scale, *F*(1, 117) = 25.28, *p* < .001, η_p_^2^ = .180.

**TABLE 3:** Scores for the ICDP and the comparison group on key measures: Mean and standard deviation before and after the ICDP course

*Variable*	*Group*	*N*	*Before ICDP M (SD)*	*After ICDP M (SD)*	*F*	*P*	η_p_^2^
Activities with the child (25–125)	ICDP	51	101.86 (11.10)	101.92 (11.71)	7.80	.006*	.08
	Comparison	43	101.95 (11.80)	98.81 (12.17)			
Positive discipline (0–105)	ICDP	91	42.81 (20.53)	50.26 (23.51)	3.52	.063	.02
	Comparison	62	44.40 (21.57)	46.56 (21.35)			
Household commotion (0–15)	ICDP	99	2.78 (2.69)	2.32 (2.45)	0.81	.370	.01
	Comparison	69	1.61 (1.95)	1.67 (2.00)			
Parenting strategy (5–30)	ICDP	117	22.67 (2.68)	23.52 (2.24)	4.82	.029*	.03
	Comparison	72	23.54 (2.83)	23.30 (2.98)			
Child management	ICDP	70	1.91 (0.37)	1.79 (0.29)	5.57	.020*	.05
	Comparison	37	1.82 (0.29)	1.83 (0.28)			
Engagement with the child	ICDP	111	2.45 (0.91)	2.21 (0.75)	1.55	.214	.01
	Comparison	67	2.09 (0.65)	2.03 (0.59)			
Emotional engagement	ICDP	111	2.44 (0.93)	2.18 (0.77)	N/A	N/A	N/A
	Comparison	67	2.08 (0.69)	2.06 (0.61)			
Strategic engagement	ICDP	121	2.86 (1.10)	2.65 (0.98)	N/A	N/A	N/A
	Comparison	71	2.54 (0.89)	2.53 (0.92)			
Happiness with partner (0–6)	ICDP	104	3.53 (0.82)	3.60 (0.85)	2.19	.141	.01
	Comparison	73	3.92 (1.04)	3.89 (0.95)			
SDQ difficulties (0–40)	ICDP	119	9.06 (4.81)	7.79 (4.65)	N/A	N/A	N/A
	Comparison	65	6.67 (3.54)	6.46 (3.81)			
SDQ prosocial (0–10)	ICDP	122	7.32 (2.13)	7.51 (2.20)	N/A	N/A	N/A
	Comparison	67	7.54 (2.32)	7.76 (2.07)			
SDQ impact score (0–10)	ICDP	117	0.51 (1.10)	0.24 (0.80)	5.71	.018*	.03
	Comparison	76	0.07 (0.30)	0.11 (0.42)			

*Notes: F* = ANOVA, interaction between Group and Time, measuring the difference in change scores between the two groups; *p* = probability; η_p_^2^ = effect size. Multivariate results for the MANOVA tests for the SDQ subscales (SDQ total difficulties and SDQ prosocial) and the subscales of the engagement scale (emotional engagement and strategic engagement) showed non-significant Group × Time interactions, respectively, *F*(386) = 0.70, *p* = .499, Wilks’ lambda = .996, η_p_^2^ = .004 and *F*(384) = 0.54, *p* = .583, Wilks’ lambda = .997, η_p_^2^ = .003. The results of the univariate ANOVA tests for SDQ total difficulties, SDQ prosocial, emotional engagement and strategic engagement are therefore not reported. *p < .05.

There was a significant interaction between Group and Time for amount of activity, indicating no change in amount of activity for the ICDP group (*M* = 101.80 and 101.92), and a decrease in activity in the comparison group (*M* = 101.95 and 98.81). Follow-up analyses confirmed a significant decline in amount of activity in the comparison group, *F*(1, 43) = 11.07, *p* = .002, η_p_^2^ = .213.

A significant interaction between Group and Time of measurement for child management reflects that ICDP caregivers reported more positive attitudes and better child management after the course (*M* = 1.91 and 1.79), while there was no change in the comparison group (*M* = 1.82 and 1.83). Follow-up analyses confirmed a significant improvement in the ICDP group on the child management scale, *F*(1, 70) = 11.94, *p* = .001, η_p_^2^ = .149.

Table 3 shows a significant interaction between Group and Time of measurement in the SDQ impact score, reflecting a decrease in parental reported overall distress and social impairment resulting from child difficulties in the ICDP group (*M* = 0.51 and 0.24), but not in the comparison group (*M* = 0.07 and 0.11). Follow-up analyses confirmed a significant improvement in reported overall distress and social impairment in the ICDP group, *F*(1, 117) = 8.06, *p* = .005, η_p_^2^ = .065.

### Initial scores as moderators of ICDP effects

Table 4 shows a significant three-way interaction between Group, Education and Time of measurement for household commotion. Caregivers with a higher education in the ICDP group showed a larger change in scores (*M* = 3.21 and 2.50) on the Confusion, Hubbub, and Order Scale than the other groups (for caregivers with a higher education in the comparison group, *M* = 1.24 and 1.49; and for caregivers without a higher education, *M* = 2.17 and 2.07 in the ICDP group, and *M* = 2.81 and 2.25 for the comparison group). Follow-up analyses confirmed a significant interaction between Group and Time of measurement for participants with a higher education but not for participants with a lower education for commotion in the household, *F*(1, 111) = 11.32, *p* = .001, η_p_^2^ = .094.

**TABLE 4:** Significant interactions between group, education/parent measures and time of measurement: Mean and standard deviation before and after the ICDP

	*Variable*	*Group*	*N*	*Before ICDP M (SD)*	*After ICDP M (SD)*	*F*	*P*	η_p_^2^
Education	Household commotion	ICDP high education	58	3.21 (2.88)	2.50 (2.59)	6.84^a^	.010*	.04
		Comparison high education	53	1.24 (1.52)	1.49 (1.67)			
		ICDP not high education	41	2.17 (2.28)	2.07 (2.24)			
		Comparison not high education	16	2.81 (2.69)	2.25 (2.82)			
Depression	Child management	ICDP low depression	32	1.82 (0.36)	1.73 (0.29)	5.25	.024*	.05
		Comparison low depression	17	1.85 (0.37)	1.80 (0.29)			
		ICDP high depression	38	1.99 (0.36)	1.84 (0.28)			
		Comparison high depression	20	1.81 (0.21)	1.86 (0.28)			
Satisfaction with social support	Household commotion	ICDP low satisfaction	45	3.29 (2.93)	2.80 (2.77)	5.76	.018*	.04
		Comparison low satisfaction	19	2.16 (2.14)	2.53 (2.50)			
		ICDP high satisfaction	36	1.89 (2.23)	1.67 (2.07)			
		Comparison high satisfaction	36	1.17 (1.75)	.97 (1.25)			
	Parenting strategy	ICDP low satisfaction	54	21.80 (2.93)	23.00 (2.46)	4.17	.043*	.03
		Comparison low satisfaction	20	23.20 (2.26)	22.15 (3.22)			
		ICDP high satisfaction	42	23.29 (2.15)	23.83 (1.91)			
		Comparison high satisfaction	36	23.94 (2.89)	24.08 (2.82)			

*Notes: F* = ANOVA, interaction between Group and Time, measuring the difference in change scores between the two groups; *p* = probability; η_p_^2^ = effect size. *p < .05.

Table 4 shows a significant three-way interaction between HADS (low/high), Group and Time for *child management.* For caregivers with low depression scores, *M* = 1.82 and 1.73 in the ICDP group, and 1.85 and 1.80 in the comparison group. For caregivers with high depression scores, *M* = 1.99 and 1.84 in the ICDP group, and 1.81 and 1.86 in the comparison group. The results indicate that participation in the ICDP course may have a greater effect on caregivers with higher depression scores. Follow-up analyses to further investigate this three-way interaction showed a significant interaction between Group and Time of measurement for parents with higher depression scores, *F*(1, 58) = 8.59, *p* = .005, η_p_^2^ = .137, but not for parents with lower depression scores. Several three-way interactions approached significance with a similar pattern. For *parenting strategy*, for caregivers with low depression scores, *M* = 23.66 and 24.16 for the ICDP group, and 24.14 and 24.11 in the comparison group. For caregivers with high depression scores, *M* = 21.95 and 23.06 in the ICDP group, and 22.97 and 22.54 in the comparison group (*F* = 2.80, *p* = .096, η_p_^2^ = .015). With regard to *commotion in the home*, for caregivers with low depression scores, *M* = 2.11 and 1.66 in the ICDP group, and 1.44 and 1.11 in the comparison group. For caregivers with high depression scores, *M* = 3.37 and 2.91 in the ICDP group, and 1.79 and 2.27 in the comparison group (*F* = 3.20, *p* = .076, η_p_^2^ = .020). Three-way interactions between depression, group and time on positive discipline, activities, engagement (strategic or emotional), and child difficulties were not significant.

There were significant three-way interactions between satisfaction with social support (low/high), group and time for parenting strategy and commotion in the home. With regard to *parenting strategy*, for caregivers with low satisfaction scores, *M* = 21.80 and 23.00 in the ICDP group, and 23.20 and 22.15 in the comparison group. For caregivers with high satisfaction scores, *M* = 23.29 and 23.83 in the ICDP group, and 23.94 and 24.08 in the comparison group. For *commotion in the home*, for caregivers with low satisfaction scores, *M* = 3.29 and 2.80 in the ICDP group, and 2.16 and 2.53 in the comparison group. For caregivers with high satisfaction scores, *M* = 1.89 and 1.67 in the ICDP group, and 1.17 and 0.97 in the comparison group. The results indicate that participating in ICDP had a more positive influence on parenting strategies and family commodity for participants who were less satisfied with their social support. Follow-up analyses showed a significant interaction between Group and Time of measurement for participants with lower satisfaction with their social support but not for participants with higher satisfaction with their social support for parenting strategy, *F*(1, 74) = 7.19, *p* = .01, η_p_^2^ = .093, and home commotion, *F*(1, 64) = 4.23, *p* = .044, η_p_^2^ = .066. There was a three-way interaction approaching significance for *child management.* For caregivers with low satisfaction scores, *M* = 2.08 and 1.87 in the ICDP group, and 1.88 and 1.91 in the comparison group. For caregivers with high satisfaction scores *M* = 1.81 and 1.72 in the ICDP group, and 1.77 and 1.74 in the comparison group (*F* = 2.86, *p* = .095, η_p_^2^ = .035). This indicates that participating in ICDP had a more positive influence on child management for caregivers who were less satisfied with their social support. Thus quality, not quantity, of social support mattered. There was no significant interaction with number of social supports. There were no three-way interactions between social support, group and time on positive discipline, activities, engagement (strategic or emotional), and child difficulties, or between scores on the Generalized Self-Efficacy Scale (low/high), group and time of measurement for measures of positive discipline, activities, engagement (strategic or emotional), parenting strategy, child management, commotion in the home and child difficulties.

## DISCUSSION

The results suggest that the ICDP may be effective in promoting positive parenting in a general community sample. There were a consistent number of positive and significant effects of the programme including parenting strategies and attitudes towards child management and perceived ability to manage the child, and social impairment resulting from child difficulties. The lack of similar changes in the comparison group indicates that the results cannot be explained by the passage of time. Several measures converged, suggesting that ICDP also may have a positive effect on the caregivers’ evaluation of child difficulties, use of positive discipline, and engagement with the child, but these trends were not significant in the interaction analysis. The comparison group scored lower on activities with the child at follow-up, whereas the ICDP group did not change. Participation in the study resulted in increased awareness of parenting influences among the participants. Both groups may have become more concerned about their parenting but only the caregivers in the comparison group scored lower because they did not receive the support that the ICDP group benefited from through the course. Another hypothesis is that participation in the ICDP course prevented some of the usual stressors on family life that may imply fewer caregiver–child interactions.

It is noteworthy that participants with low initial scores in particular seemed to benefit from participation in the programme. Caregivers may have different reasons for taking part in the programme. Some caregivers may look for a parenting programme because they are struggling with everyday child rearing and are lacking useful strategies, because it has been suggested by a friend or a teacher in the kindergarten, or they may be referred to the programme by social services. The fact that there were more single parents in the ICDP group may reflect the increased strain of childrearing with one rather than two parents. Other studies of parenting programmes also report a relatively large proportion of single parents (Almeida et al., 2012). It is in line with the aims of the programme that parents who seem to struggle most, show the greatest positive change. Other caregivers might have good self-confidence and experience few problems with their children, and attend the programme because they want all the knowledge they can get. This group shows less change because participation in the programme is consolidating their existing attitudes and use of strategies rather than initiating change.

One result of the broad focus of the ICDP approach is differential effect on various subgroups. There was a decrease in commotion in the home following ICDP participation but this was only significant for caregivers with higher education. Other studies of parenting programmes have also found that parents with lower education may benefit less than more educated parents (Almeida et al., 2012; Fossum, Drugli, Handegård, & Mørch, 2010), but in this study the moderating effect of education applied only to this domain. Participation in the programme seemed to lead to a larger positive change in attitudes towards child management and perceived ability to manage the child in caregivers with relatively higher depression scores on HADS. Caregivers who expressed less satisfaction with the social support they received showed greater improvement in parenting strategies and lower commotion in the home than caregivers who were more satisfied with their social support. Several studies of parenting programmes have focused on caregivers who are clinically depressed or at severe social risk (Boyd & Gillham, 2009; Law, Plunkett, Taylor, & Gunning, 2009). A community-wide programme may contribute to positive parenting in mothers and fathers with subclinical depression who are not usually referred to the mental health services. Several authors have pointed to a need for generally available parenting programmes (Sanders & Morawska, 2010; Shapiro, Prinz, & Sanders, 2008) such as this programme, which appears to reach caregivers in need of support and advice who may experience the task of raising children as manageable but challenging, but have no diagnosis or clear clinical challenge.

There are some limitations with the present study. All children in both groups were of preschool age, yet there was a variation in the ages. There were insufficient subgroups to break down the results by age, but this may be an important factor in subsequent studies. Another limitation was the fact that there were some significant differences between the ICDP and comparison group at baseline and hence they do not necessarily represent the same population. The comparison group was recruited from health centres and kindergartens where ICDP courses were not available, while the ICDP group seems to have made some self-initiated or other-initiated efforts to follow the programme. This means that the ICDP group to some extent might have been biased and have had more motivation and potential for change than the comparison group. However, this is not only a weakness. Most evaluations are of programmes implemented by research institutions (Rodrigo et al., 2012). The present study gives evidence that ICDP (or other general parenting programmes) may reach the group it is aiming at and function in community-wide implementation. Other limitations are attendance and loss to follow-up by approximately half of the participants, which may have skewed the results for successful participants; those with more time, support or less depression being overrepresented at follow-up. Baseline comparisons showed that those with partners and lower depression scores were more likely to respond. Question omission indicated by the variation in N may have resulted in reduced numbers and diminished power. Finally, caution should also be exerted as a result of the multiple F-tests conducted (Bakan, 1969) and self-reported measures.

A pre-investigation was conducted to address the quality of implementation, and the relationship between number of meetings attended and change scores was addressed. Future investigations should address the relationship between implementation quality and programme effects more specifically. There is still a lack of knowledge about mediating processes and the interaction between programme features and child and parent characteristics (Dekovic, Stoltz, Schuiringa, Manders, & Asscher, 2012; Law et al., 2009). Some programme features may benefit most caregivers while other features may be beneficial for parents and children with particular characteristics, even if clinical groups are excluded. Mechanisms accounting for change are not fully understood and may include group conversation, regulation without strict control or new skills. A larger randomized controlled trial could shed more light on this, as would additional observations (Davé, Nazareth, Senior, & Sherr, 2008), and longer term outcomes (Sandler et al., 2011).

The results of this and other studies indicate that caregivers may benefit from participating in parenting programmes, including caregivers (and children) without clinical conditions. The results support the call for community-wide implementations of ICDP and other parenting programmes in spite of the heterogeneous nature of the population (Rodrigo et al., 2012). The basic philosophy of ICDP, with a focus on positive emotion and regulation, rather than on control, which is more apparent in parenting programmes for parents who have children with behaviour disorders, may resonate well in many parents who experience the ordinary challenges of everyday child-rearing, and who may not need or feel comfortable with a more controlling approach.
